# Using imputed pre-treatment cholesterol in a propensity score model to reduce confounding by indication: results from the multi-ethnic study of atherosclerosis

**DOI:** 10.1186/1471-2288-13-81

**Published:** 2013-06-21

**Authors:** Neal W Jorgensen, Christopher T Sibley, Robyn L McClelland

**Affiliations:** 1 Department of Biostatistics, University of Washington, Seattle, WA, USA; 2 National Institutes of Health Clinical Center, Bethesda, MD, USA

**Keywords:** Multiple imputation, Confounding by indication, Propensity score, Inverse probability of treatment weights, Statins

## Abstract

**Background:**

Studying the effects of medications on endpoints in an observational setting is an important yet challenging problem due to confounding by indication. The purpose of this study is to describe methodology for estimating such effects while including prevalent medication users. These techniques are illustrated in models relating statin use to cardiovascular disease (CVD) in a large multi-ethnic cohort study.

**Methods:**

The Multi-Ethnic Study of Atherosclerosis (MESA) includes 6814 participants aged 45-84 years free of CVD. Confounding by indication was mitigated using a two step approach: First, the untreated values of cholesterol were treated as missing data and the values imputed as a function of the observed treated value, dose and type of medication, and participant characteristics. Second, we construct a propensity-score modeling the probability of medication initiation as a function of measured covariates and estimated pre-treatment cholesterol value. The effect of statins on CVD endpoints were assessed using weighted Cox proportional hazard models using inverse probability weights based on the propensity score.

**Results:**

Based on a meta-analysis of randomized controlled trials (RCT) statins are associated with a reduced risk of CVD (relative risk ratio = 0.73, 95% CI: 0.70, 0.77). In an unweighted Cox model adjusting for traditional risk factors we observed little association of statins with CVD (hazard ratio (HR) = 0.97, 95% CI: 0.60, 1.59). Using weights based on a propensity model for statins that did not include the estimated pre-treatment cholesterol we observed a slight protective association (HR = 0.92, 95% CI: 0.54-1.57). Results were similar using a new-user design where prevalent users of statins are excluded (HR = 0.91, 95% CI: 0.45-1.80). Using weights based on a propensity model with estimated pre-treatment cholesterol the effects of statins (HR = 0.74, 95% CI: 0.38, 1.42) were consistent with the RCT literature.

**Conclusions:**

The imputation of pre-treated cholesterol levels for participants on medication at baseline in conjunction with a propensity score yielded estimates that were consistent with the RCT literature. These techniques could be useful in any example where inclusion of participants exposed at baseline in the analysis is desirable, and reasonable estimates of pre-exposure biomarker values can be estimated.

## Background

Randomized controlled clinical trials (RCTs) are a common and useful way to study the effects of medications on outcomes. Randomization ensures that participants receiving different treatments are comparable. However, randomized trials cannot address every possible question of interest. Observational studies often have different endpoints (e.g. advanced magnetic resonance imaging of heart size and function) that may not have been studied in an RCT, longer follow-up, unique populations, or some other inherent practical advantage for examining certain associations. This leads to the challenge of studying medication effects on endpoints in the observational setting, where issues of confounding by indication are challenging to deal with. Participants that receive the medication in practice differ systematically from those who do not in terms of outcome risk factors. In the case of statins, a widely prescribed cholesterol lowering medication [1], treated participants would on average be those with higher underlying cholesterol levels and greater cardiovascular risk factor burden.

An approach to this issue is to use a “new-user” design, whereby prevalent users of medications at baseline are excluded. The new-user design has important advantages such as eliminating bias from time-varying hazards that arise when including prevalent users. Additionally, the new-user design allows the pre-treatment participant characteristics to be used in the analysis to control for confounding [2]. However, there are several situations where it is advantageous or even necessary, to include prevalent users in the analysis. For example, the number of participants taking medication already may be very large, and exclusion of this subset may be prohibitive in terms of sample size and power. Another example is the situation where the endpoint of interest is only measured at one exam, often the baseline exam, and a cross-sectional analysis including prevalent users is the only choice.

A common technique to deal with confounding by indication in observational studies is to use a propensity score. Propensity scoring is a method of obtaining a summary score that can be used to control for a collection of confounding variables in a study [3]. A propensity score for a particular exposure is the conditional probability that a person will be exposed given a set of observed covariates. In principle, the effect of the exposure can then be measured among participants who have the same predicted propensity of treatment, thus controlling for the confounding [4]. This has been shown to be effective at reducing indication bias in other observational studies [5].

An assumption of the propensity score approach is that all confounding variables (influencing both the exposure and the outcome) have been measured. In the setting of an observational cohort study this is a problematic assumption when using exposure at study baseline. The underlying value of the biomarker (e.g. cholesterol) is often a primary determinant of exposure (e.g. statins), and for those exposed (e.g. taking statins) the underlying value is not measured. Thus, the propensity score will not correctly eliminate confounding due to the biomarker. To deal with this issue, we consider the underlying pre-treatment cholesterol levels from the subset of participants on lipid-lowering medications at baseline as “missing” data, then following the techniques by McClelland et al. [6], impute the pre-treatment cholesterol levels as a function of observed on-treatment cholesterol levels, medication type, and dose. Multiple imputation techniques are used to incorporate the variability induced by the estimation. The treated cholesterol values are then replaced with the imputed pre-treatment cholesterol values for those on lipid-lowering medication. The propensity score is estimated using this new cholesterol variable, in addition to other covariates. This modified propensity score is then used to reduce the indication bias in a Cox proportional hazard model for time to incident cardiovascular disease (CVD) endpoints where time starts at entry into the study and where participants are free of CVD at entry. This is an example where the effects of statins have been extensively studied in numerous RCTs.

## Methods

### The Multi-Ethnic Study of Atherosclerosis (MESA) data

MESA is a prospective cohort study of the prevalence, risk factors and progression of subclinical cardiovascular disease in a multi-ethnic community-based cohort. The study includes 6814 participants with no known CVD at baseline, aged 45-84 years who identified themselves as White, African-American, Hispanic, or Chinese recruited from six United States communities between 2000 and 2002. The communities were Forsyth County, North Carolina; Northern Manhattan and the Bronx, New York; Baltimore City and Baltimore County, Maryland; St. Paul, Minnesota; Chicago, Illinois; and Los Angeles County, California. Each site recruited an approximately equal number of men and women, according to pre-specified age and race/ethnicity proportions. All participants gave informed consent. Details of the sampling, recruitment, and data collection have been reported elsewhere [7]. Blood lipid measurements were obtained following an overnight fast. Low-density lipoprotein (LDL) cholesterol was calculated in plasma specimens having a triglyceride value of *<*400 mg*/*dL using the formula of Friedewald et al. [8]. Medication use was determined by a questionnaire. The participant was asked to bring to the clinic containers for all medications used during the two weeks prior to the visit. The interviewer then recorded the name of each medication, the prescribed dose, and frequency of administration from the containers. MESA is an ongoing, multi-faceted study, with results spanning many areas of research.

CVD was ascertained at intervals of 9-12 months by a telephone interviewer who contacted each participant to inquire about interim hospital admissions and cardiovascular outpatient diagnoses. Trained personnel abstracted medical records suggesting possible cardiovascular events, and two physicians independently classified the events. The endpoints included in this analysis were incident hard coronary heart disease (CHD) and incident hard CVD. Hard CHD is defined as definite or probably myocardial infarction (MI) or CHD death. Hard CVD is defined as hard CHD or stroke. Definite or probable MI required either abnormal cardiac biomarkers; evolving Q waves; or a combination of chest pain, characteristic ECG changes, and abnormal biomarker levels. Fatal CHD required a documented MI within 28 days of death, chest pain within 72 hours of death, or a history of CHD without a known non-cardiac cause of death.

### Imputation model

The first step in the imputation process is to identify a subset of participants that have both pre- and post-treatment (lipid-lowering medication) measurements of cholesterol. These are the participants who are not on lipid-lowering medication at baseline and then go on to start medications at subsequent visits. We refer to this subset as the new-user cohort and there are N = 1286 participants that qualify. The new-user cohort is used to establish a linear regression relationship between pre-treatment cholesterol values and post-treated cholesterol values, medication data and relevant participant characteristics. The variables ultimately chosen for this regression model are then used to impute pre-treatment cholesterol levels for participants on lipid-lowering medication at baseline. Note that unlike traditional missing data imputation approaches (in which the missing cholesterol of those on treatment would be imputed based on the observed cholesterols of other participants at the same exam who were untreated), we are using an independent subset of new-users with pre- and post- treatment values to develop the imputation model. In the absence of such a subset, one could use medication effects reported in the meta-analysis literature to estimate pre-treatment values [6].

Post-treatment cholesterol is important in explaining the variability of the pre-treated cholesterol. In addition, other participant characteristics such as age, gender, race/ethnicity and risk factors, and the medication type and dose are also important. This analysis is principally interested in the relationship of statins with CVD as they were the most commonly prescribed lipid-lowering medication in this population. We observed use of six types: atorvastatin, fluvastatin, lovastatin, pravastatin, simvastatin, and rosuvastatin. Other, non-statin, drugs with lipid modifying effects were used as covariates to impute underlying cholesterol levels. The non-statin group was categorized into 4 subclasses, resins, fibrates, niacin, and ezetimibe. Indicator variables were created for each type of statin and for each of the subclasses of non-statins. Participants on multiple medications and those taking combination medications were assigned indicators for each medication. Medication dosage was acquired from the prescribed dose (in milligrams) written on the medication containers, and medication indicators were assigned a value from 0-2 where 0 = not on drug, 1 = on drug, low dose, and 2 = on drug, high dose. Those prescribed ≥40 mg per day of a given statin (or ≥20 mg per day of atorvastatin) were considered to be on high dose. When the high dose group had less than 10 participants it was collapsed down to a binary yes/no variable.

From the full MESA cohort (6814) there were 1086 participants (16%) on lipid-lowering medications at baseline. We refer to this group as the baseline-user cohort and they are the ones for which pre-treatment cholesterol will be imputed. In order for the imputation to be carried out easily using existing software, the new-user and baseline-user cohort data were stacked as illustrated in Table 1. All the pre-treated cholesterol levels will be missing for the baseline-user cohort while the new-user cohort will have all non-missing data, corresponding to the observed cholesterol at the exam before they commenced taking a lipid-lowering medication. Note that we are stacking data from different exams for convenience, since they all represent pre-treatment values. Similarly, the post-treatment values and covariates are stacked, and the new-user observations correspond to the exam immediately after starting lipid lowering medication. For the baseline-user cohort the post-treatment value is always present and is their observed cholesterol at baseline, and covariates are also all taken from baseline. Standard imputation commands can then be used to generate the imputations. Once the imputations are constructed, we then merge these imputed values back in with the baseline data as the pre-treatment cholesterol value for those on medication at baseline (e.g. for the 1086 in the baseline-user cohort). For the remaining participants not on medication the original observed cholesterol is used. In previous work by McClelland *et al.*[6] it was shown that in MESA baseline statins users and new statin users were comparable in terms of age, gender, race, body mass index, smoking, diabetes status, and type and dose of lipid lowering drug. The new medication users tended to have a higher rate of hypertension (67.6 *versus* 62.3 per cent, *p* = 0*.*05) and tended to have treated LDL cholesterol an average of five units lower (99.5 *versus* 104.5 mg*/*dL, *p* = 0*.*002).

**Table 1:** Structure of the dataset used for imputation

**Observations**	**Y = Pre-treated cholesterol**	**X = Post-treated cholesterol**	**Z = Covariates**
New lipid-lowering medication users (N = 1286)	Exam dependent cholesterol levels (exam prior to starting lipid-lowering medication)	Exam dependent cholesterol levels (exam after starting lipid-lowering medication)	Exam dependent covariates (exam after starting lipid-lowering medication)
Lipid-lowering medication users at baseline (N = 1086)	Missing	Post-treated cholesterol from exam 1	Baseline covariates from exam 1

Multiple imputation techniques following the algorithm of Van Buuren et al. [9] as implemented and described by Royston [10,11] are used to incorporate the extra variability induced from the imputation. Essentially, rather than impute one single pre-treatment cholesterol level for participants on lipid-lowering medication we generate 10. For each realization, the corresponding set of complete data is analyzed in a standard manner and the results are pooled using a set of rules proposed by Rubin [12]. The imputation method regresses pre-treated cholesterol with post-treated cholesterol and specified covariates and then random draws are taken from the conditional distribution of the missing cholesterol variable given the post-treated cholesterol and covariates. McClelland *et al.*[6] showed that this multiple imputation technique yields more realistic parameter estimates than other traditional approaches to missing data. Imputation is performed with Stata 11.2 using the *ice* (imputation by chained equations) routine written by Royston [11].

An assumption in multiple imputations is that of missing at random (MAR) [13]. Essentially, MAR means the probability of missingness may depend on the data that was observed but not on data values that are missing [14]. In our case the missingness in pre-treated cholesterol was predetermined based on whether or not that participant was on lipid-lowering medication at baseline.

### Propensity score model

The propensity score is obtained from a logistic regression model and is the conditional probability of being on treatment given a set of covariates: e(*X*) = Pr(*Y* = 1|*X*), where *X* = cholesterol, clinic site, age, gender, race/ethnicity, body mass index, systolic and diastolic blood pressures, heart rate, hypertension status, hypertension medication use, smoking status, pack-years of cigarette smoking, alcohol use, family history of MI, diabetes, education, income, health insurance, marital status, general health, depression, intentional exercise, coronary artery calcification (CAC), common and internal carotid intimal-medial thickness (IMT), creatinine, fibrinogen, and high sensitivity C-reactive protein (hs-CRP). CAC and IMT were natural log transformed and continuous variables were centered about their means. Estimated propensity scores were created for each of the 10 imputations. For comparison, a propensity score was also created following a new-user design [2] where prevalent users at exam 1 are excluded and the propensity is based on those who start statins at exam 2.

Stabilized inverse probability of treatment weights (SW) were created for each of the propensity scores to be used in the weighted regression models. Inverse probability weighting can be used to estimate the exposure effects by appropriately adjusting for confounding and selection bias [15]. The weighting creates a pseudo-population in which the exposure is independent of the measured confounders. In such a pseudo-population one can regress the outcome on the exposure using conventional regression techniques [16]. We use stabilized inverse probability weights as suggested by Xu *et al.*[17] who found that it’s possible to preserve the sample size in the pseudo-population close to the original data and produce a reasonable estimation of the variance of the main effect while maintaining an appropriate type I error rate. SWs are defined as SW= $\frac{p}{\hat{e}\left(X\right)}$ for those on statins and $\frac{1-p}{1-\hat{e}\left(X\right)}$ for those not on statins, where $\hat{e}\left(X\right)$ = estimated propensity score and *p* = observed proportion of statin users. In order to limit the influence of extreme weights, the SWs were set to .10 for weights less than .10 and to 10 for weights greater than 10 [18]. For sensitivity, non-truncated weights were also generated as well as different thresholds for truncating.

### Endpoint models

We use weighted Cox proportional hazard models with SWs to estimate the exposure effect of statins on CVD endpoints using robust standard errors. For each CVD endpoint we generate four separate Cox models. One model will be unweighted and adjusted for traditional cardiovascular risk factors (TRF), while the others will utilize SWs from different propensity model specifications. One specification does not include any cholesterol adjustment, one adjusts for observed cholesterol for those not on lipid-lowering medications at baseline and imputed pre-treatment cholesterol for those on lipid-lowering medications at baseline. Additionally, a new-user design is also implemented where prevalent statin users are excluded and weights derived from the propensity of being a new-user of statins at exam 2. Multiple imputation techniques are used for the Cox models that use weights based on the imputed cholesterol. Kaplan-Meier plots were generated to illustrate the differences across weighting schemes and models.

## Results

Participants who had non-missing CVD and covariate data were included (N = 6035) in the endpoint analysis. The mean age of included participants was 62 years (range 45-84): 53% were women, 39% were Caucasian, 26% were African-American, 22% were Hispanic, and 13% were Chinese American. To be consistent with the RCT literature on statin effects, follow-up for the CHD analysis was truncated at 5-years. During this time period there were 119 incident hard CHD events and 190 incident hard CVD events. After excluding participants with missing covariate data there were 968 (16%) participants taking lipid lowering medication at baseline, of which 885 were taking a statin. As anticipated, participants taking statins had more adverse CVD risk factors at baseline and a higher burden of subclinical calcified atherosclerosis than those not taking statins (Table 2).

**Table 2:** Participant characteristic by baseline statin use

	**Baseline statin use**					
	**Non-weighted**			**Weighted using stabilized IPTW**^**1**^		
**Characteristics**	** No (N = 5150)**	** Yes (N = 885)**		** No (N = 5150)**	** Yes (N = 885)**	
	**Mean (SD) or n (%)**	**Mean (SD) or n (%)**	**P-value**	**Mean (SD) or n (%)**	**Mean (SD) or n (%)**	**P-value**
Age, Mean (SD), y	61.3 (10.3)	65.9 (8.8)	<.001	62.0 (10.2)	62.9 (9.7)	0.141
Male	2441 (47.4%)	417 (47.1%)	0.879	2530 (47.7%)	401 (50.8%)	0.308
Race/ethnicity						
White	1976 (38.4%)	385 (43.5%)	0.004	2069 (39.0%)	351 (44.5%)	0.156
Chinese	672 (13.1%)	99 (11.2%)		650 (12.3%)	74 (9.4%)	
Black	1323 (25.7)	235 (26.6%)		1355 (25.6%)	186 (23.6%)	
Hispanic	1179 (22.9%)	166 (18.8%)		1226 (23.1%)	178 (22.6%)	
Body mass index, kg/m^2^	28.1 (5.5)	28.7 (5.0)	<.001	28.2 (5.4)	28.5 (5.2)	0.235
Current smoker	690 (13.4%)	83 (9.4%)	0.001	659 (12.4%)	84 (10.6%)	0.394
Pack years smoking	11.0 (22.3)	12.9 (22.4)	0.021	11.1 (22.2)	10.9 (21.5)	0.880
Current alcohol use	2902 (56.4%)	507 (57.3%)	0.603	2997 (56.5%)	479 (60.7%)	0.128
Diabetes	534 (10.4%)	191 (21.6%)	<.001	729 (13.8%)	(111 (14.0%)	0.897
Family history of MI	1952 (37.9%)	427 (48.3%)	<.001	2153 (59.4%)	334 (42.4%)	0.542
Hypertension	2082 (40.4%)	543 (61.4%)	<.001	2301 (43.4%)	358 (45.3%)	0.492
Hypertension Meds	1645 (32.0%)	520 (58.8%)	<.001	1941 (36.6%)	309 (39.2%)	0.323
Total cholesterol, mg/dL	196.7 (35.7)	224.1 (36.4)	<.001	206.4 (50.9)	204.4 (41.2)	0.611
Systolic BP, mm Hg	125.5 (21.3)	129.7 (21.6)	<.001	126.1 (21.1)	127.3 (22.3)	0.286
Diastolic BP, mm Hg	72.0 (10.4)	71.2 (9.6)	0.038	71.9 (10.3)	72.0 (9.9)	0.727
Health insurance	4644 (90.2%)	861 (97.3%)	<.001	4789 (90.4%)	741 (93.9%)	0.109
Income						
<$25 K	1586 (30.8%)	285 (32.2%)	0.165	1679 (31.7%)	229 (29.0%)	0.592
$25 K-$49 K	1492 (29.0%)	250 (28.3%)		1558 (29.4%)	237 (30.0%)	
$50 K-$99 K	1375 (26.7%)	212 (24.0%)		1351 (25.5%)	198 (25.1%)	
$100 K+	697 (13.5%)	138 (15.6%)		712 (13.4%)	125 (15.9%)	
Education						
< High school	914 (17.8%)	143 (16.2%)	0.164	941 (17.8%)	124 (15.7%)	0.381
High school	2352 (45.7%)	420 (47.5%)		2475 (46.7%)	366 (46.4)	
College	937 (18.2%)	142 (16.1%)		914 (17.3%)	124 (15.8%)	
Graduate school	947 (18.4%)	180 (20.3%)		970 (18.3%)	174 (22.1%)	
Married	3160 (61.4%)	546 (61.7%)	0.850	3246 (61.3%)	502 (63.6%)	0.406
Intentional exercise (met-min/wk)	1583 (2396)	1527 (2130)	0.480	1644 (2421)	1692 (2477)	0.705
Creatinine, mg/dL	0.94 (0.23)	1.01 (0.41)	<.001	0.97 (0.31)	0.97 (0.30)	0.778
Fibrinogen, mg/dL	342.6 (72.8)	357.4 (74.6)	<.001	351.3 (83.8)	356.4 (88.0)	0.530
hs-CRP, mg/L	3.7 (5.8)	3.2 (4.8)	0.004	3.6 (5.4)	4.2 (7.3)	0.396
CAC (Agatston)	122 (367)	240 (522)	<.001	149 (400)	161 (444)	0.525
Common carotid (mm)	0.86 (0.19)	0.91 (0.19)	<.001	0.87 (0.19)	0.88 (0.19)	0.195
Internal carotid (mm)	1.03 (0.57)	1.25 (0.67)	<.001	1.08 (0.61)	1.13 (0.66)	0.139
Heart rate (bpm)	63.0 (9.5)	63.6 (9.8)	0.066	63.3 (9.8)	63.8 (10.4)	0.455

^1^ Comparisons for the first imputation.

Table 3 contains the parameter estimates for the imputation model. The model has an adjusted R^2^ = 0.44. Based on this model 10 imputations of pre-treatment cholesterol were created for each participant in the baseline-user cohort. On average the imputed pre-treatment total cholesterol was 39.1 mg/dL(SD = 34.2) higher than the measured post-treatment value. Imputation of pre-treatment high-density lipoprotein (HDL-c) demonstrated only a small treatment related shift. Rather than introduce the additional variability we used observed HDL-c values for the remainder of the analysis.

**Table 3:** Parameter estimates from the linear regression model for total cholesterol

**Adjusted R**^**2**^ **= 0.44**	**Cholesterol**_**pre-treated **_**(N = 1286)**		
**Covariates**	***β***	**(95% CI)**	**P-value**
Cholesterol_post-treated_ (mg/dl)	0.53	(0.46,0.60)	<.001
Age (years)	-0.18	(-0.35,-0.01)	0.033
Gender – Male	-10.32	(-13.66,-6.98)	<.001
Race/ethnicity			
White	--	--	--
Chinese	-6.21	(-11.71,-0.72)	0.027
Black	-7.62	(-11.63,-3.60)	<.001
Hispanic	-1.21	(-5.32,2.90)	0.565
Diabetes			
Normal	--	--	--
IFG	0.23	(-3.80,4.26)	0.910
Untreated diabetes	2.69	(-9.46,14.85)	0.664
Treated diabetes	-9.00	(-12.91,-5.10)	<.001
Hypertension Meds – Yes	-5.43	(-8.79,-2.08)	0.002
HDL_post-treated_	0.12	(-0.03,0.26)	0.107
triglycerides	0.03	(-0.00,0.05)	0.069
Atorvastatin			
Yes – Low dose	28.31	(18.19,38.42)	<.001
Yes – High dose	42.84	(31.99,53.69)	<.001
Fluvastatin			
Yes – Low dose	18.24	(1.01,35.47)	0.038
Yes – High dose	25.83	(13.15,38.51)	<.001
Lovastatin			
Yes – Low dose	19.41	(8.61,30.21)	<.001
Yes – High dose	34.80	(20.87,48.74)	<.001
Pravastatin			
Yes – Low dose	20.23	(8.69,31.77)	0.001
Yes – High dose	23.72	(8.71,38.73)	0.002
Simvastatin			
Yes – Low dose	45.46	(22.98,67.94)	<.001
Yes – High dose	19.16	(-16.64,54.96)	0.294
Simvastatin*Cholesterol_post-treated_ Interaction			
Yes – Low dose	-0.12	(-0.25,0.01)	0.071
Yes – High dose	0.11	(-0.11,0.32)	0.320
Rosuvastatin – Yes	38.36	(26.43,50.29)	<.001
Fibrate – Yes	7.00	(-3.76,17.77)	0.202
Resin – Yes	-7.76	(-31.48,15.96)	0.521
Niacin – Yes	-29.12	(-56.25,-2.00)	0.035
Niacin*Cholesterol_post-treated_ Interaction	0.12	(-0.02,0.27)	0.101
Ezetimibe – Yes	12.10	(2.27,21.94)	0.016

The propensity models were evaluated by comparing the balance of the covariates between statin users at baseline both before and after weighting. All the covariates after weighting were balanced as is shown in Table 2 for the first imputation. When comparing the baseline total cholesterol levels, new-users (n = 471) had a mean of 220.1 mg/dL. (sd = 35.4) and we estimated the pre-treated total cholesterol levels for the prevalent-users (n = 888) as 224.0 mg/dL. (sd = 24.7). This suggested that once we account for statin therapy, the two groups are not greatly different in the level of dyslipidemia at baseline.

From a meta-analysis of 9 statin RCTs with over 47,000 subjects with and without prior clinical cardiovascular disease Cheung et al. [19] found that statins are associated with a reduced risk of CHD with an estimated relative risk ratio of 0.73 (95% confidence interval (CI): 0.70, 0.77). There was a relatively consistent protective trend in other meta-analyses of statin RCTs despite the varying demographics and different definitions of CVD endpoints. For example, Thavendiranathan et al. [20] used only CVD free participants from 7 trials (n = 42,848) and found the RR of major coronary events and revascularization to be 0.71. The Cholesterol Treatment Trialists’ (CTT) Collaborators [21] looked at 27 randomized trials (n = 134,537) and observed a 0.79 reduction in major vascular events (non-fatal MI or coronary death), strokes, or coronary revascularizations. Kostis et al. [22] looked at 18 trials (n = 141,235) with sex-specific outcomes and various definitions of CVD endpoints and found women and men had similar statin effects at 0.81 and 0.77 respectively.

We analyzed the relationship between statin use and time to CVD endpoints using Cox proportional hazard models, which are summarized in Table 4. In an unweighted model adjusted for TRFs statins appear to have little association with CVD risk, with a Hazard Ratio (HR) = 0.97, 95% CI: 0.60, 1.59. Using stabilized weights from a propensity model that did not include any cholesterol adjustment the HR was slightly more protective (HR = 0.92, 95% CI: 0.54-1.57) though still closer to the null than expected. The results using the new-user design showed a similar association (HR = 0.91, 95% CI: 0.45-1.80). When stabilized weights were used from a propensity model that included imputed pre-treatment cholesterol we found the HRs for statins to be protective of CHD with a HR = 0.74, 95% CI: 0.38, 1.42.. These results are consistent in direction and magnitude from what was found by Cheung [19] and others in the meta-analyses of statins.

**Table 4:** Hazard ratios for statins from cox proportional hazards models for incident CHD

	**CHD, Hard**		**CVD, Hard**	
	**(Events = 119)**		**(Events = 190)**	
**Model (N = 6035)**	**HR (95% CI)**	**p-value**	**HR (95% CI)**	**p-value**
**Unweighted**^**1**^	0.97 (0.60,1.59)	0.915	0.92 [0.62,1.36]	0.676
**Weighted**				
**Propensity model**				
No cholesterol	0.92 [0.54,1.57]	0.767	0.82 [0.54,1.26]	0.373
Imputed cholesterol^2^	0.74 [0.38,1.42]	0.363	0.72 [0.43,1.21]	0.215
New-user analysis (N = 4886)	0.91 [0.45,1.80] (Events = 100)	0.777	0.71 [0.38,1.30] (Events = 153)	0.265
Sensitivity analysis^3^				
No truncating	0.59 [0.27,1.32]	0.199	0.62 [0.34,1.15]	0.130
<.2 & >20	0.68 [0.34,1.36]	0.275	0.69 [0.40,1.17]	0.166
<.3 & >30	0.64 [0.30,1.34]	0.238	0.66 [0.38,1.16]	0.152
<.3 & >10	0.74 [0.39,1.42]	0.370	0.73 [0.44,1.21]	0.220

^1^ Adjusted for traditional risk factors: Age, gender, race/ethnicity, BMI, diabetes status, family history of heart attack, smoking status, hypertensive medications, systolic BP.

^2^ Multiple imputations were used for models where imputed cholesterol was used in creating the weights. Weights < .1 were set to 0.1 and weights >10 were set to 10.

^3^ Using non-truncated stabilized weights and various thresholds for truncating.

There were between 4-8 participants that had their weights truncated for each of the 10 imputations using the < .1 and >10 thresholds. For sensitivity to truncating and chosen thresholds we also looked at using the non-truncated weights and various thresholds. We found that when using the non-truncated weights there was a shift in the HR from 0.74 to 0.59. The choice of thresholds affected the estimates less with HRs ranging from 0.64 to 0.74 with a range of 4 to 179 participants being truncated for the various thresholds.

**Figure 1:**
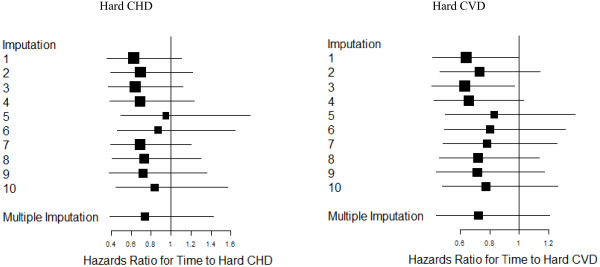
Hazard ratios from cox proportional hazards models with multiple imputations by CVD endpoint.

**Figure 2:**
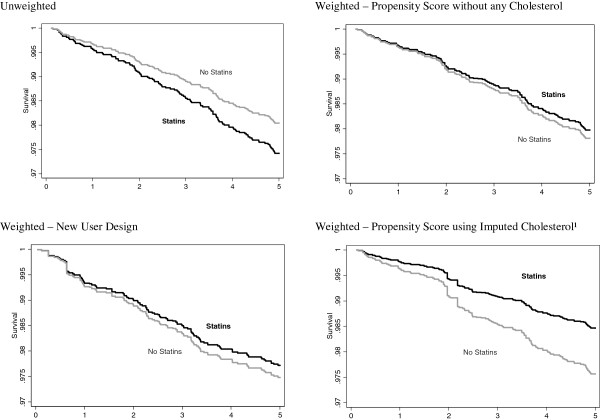
Kaplan-Meier survival curves for hard CHD.

Figure 1 contains a plot of the hazard ratios for statins by endpoint for each of the imputations. The point estimates are consistent in sign and magnitude across the imputations and the within-imputation variability appears to be greater than the between imputation variability. In Figure 2 we illustrate the effects of the various weighting schemes with Kaplan-Meier plots [23]. The first imputation was used for illustration purposes when imputed values were used. This imputation was representative of the general pattern across imputations. The proportion free of incident CHD appears on the y-axis, with separate curves for statin users and non-users at baseline. In the unweighted version, statin users appear to be at increased risk of CHD. If one weights using traditional risk factors other than cholesterol or weights based on the new-user design then we observe a slightly protective effect of statins on CHD. When the weights are based on the imputed pre-treatment cholesterol there is a clear protective association of statin use on CHD consistent with previous RCTs.

## Discussion

When studying the effects of medications in the setting of an observational study it is extremely important to reduce bias to the greatest extent possible. The assumption of no unmeasured confounders is essential to techniques such as propensity score reweighting. In the setting of observational cohort studies, this assumption is not satisfied if one intends to make use of the baseline data. A common technique to deal with this is the new-user design [2]. The new-user design has limitations and may not always be possible. For example, there will be a decrease in sample size and power and depending on the percentage of prevalent medication users this could prove problematic. In our new-user analysis our sample size decreased by over 1000 participants and 37 CVD events. This will pose an even larger problem when medications are more prevalent than what was observed for statins in MESA. To illustrate, in MESA nearly half the participants are prevalent anti-hypertensive medication users at baseline so excluding this subset would drastically cut sample size and power. Furthermore, the first exam in a longitudinal observational study is often more extensive and certain costly or exploratory measures may only be available at that exam. If the outcome of interest is only available at the baseline exam then a new-user design is not possible and one would be required to utilize prevalent users in order to answer the question of interest.

In this paper we have restricted attention to statin use at baseline as the exposure of interest. In the models for incident CVD we could have updated exposure status over time, however, this would have induced some complexity that is not central to the methods described here. That is, a second source of confounding known as time dependent confounding would have to be considered. In basic terms, this occurs when the exposure (e.g. statin use in our example) varies over time, and there is a biomarker (e.g. cholesterol) that is both a predictor of future exposure use (making it a confounder), and on the causal pathway between past exposure and outcome (making it a mediator). Simple adjustment for the biomarker at each time point handles the confounding, but adjusts out a large part of the medication effect. Traditional approaches to handling this problem include G-estimation and marginal structural models (MSM) [24,25]. There is no reason in principal that the imputed pre-treatment cholesterol for baseline users could not be incorporated into the inverse probability of treatment weights at the baseline exam in an MSM if baseline medication users were to be included.

For this paper we have restricted our analysis to the continuous pre-treatment risk factor variable cholesterol. Further research will be necessary to address how the imputation and outcome models would be affected if the pre-treatment risk factor is categorical or binary.

## Conclusions

In our examples we illustrated that traditional approaches to reducing indication bias such as standard propensity score weighting [5] and new-user designs [2] produced effect estimates for statins that were attenuated in terms of coronary heart disease risk. The imputation of pre-treated cholesterol levels for participants on lipid-lowering medication at baseline in conjunction with a propensity score yielded estimates that were consistent with the meta-analysis literature and which appeared to be much more successful at mitigating the indication bias and the effects of unmeasured confounding. These techniques could be useful in any example where inclusion of participants exposed at baseline in the analysis is desirable, and reasonable estimates of pre-exposure biomarker values can be estimated.

## Competing interests

The authors declare that they have no competing interests.

## Authors’ contributions

RML conceived the study and NWJ performed the statistical analysis and prepared the manuscript, including figures and tables. All authors participated in the interpretation and critical revision of the manuscript and all authors read and approved the final manuscript.

## Pre-publication history

The pre-publication history for this paper can be accessed here:

http://www.biomedcentral.com/1471-2288/13/81/prepub
